# Indoor Application of Coupled FLOCponics System with Caipira Lettuce (*Lactuca sativa*) Affects the Growth Performance and Water Characteristics of Far Eastern Catfish (*Silurus asotus*) and Tropical Eel (*Anguilla bicolor*)

**DOI:** 10.3390/ani15152305

**Published:** 2025-08-06

**Authors:** Jun Seong Park, Hae Seung Jeong, Jeong-ho Lee, Ju-ae Hwang

**Affiliations:** 1 Advanced Aquaculture Research, National Institute of Fisheries Science, Changwon 51688, Republic of Korea; pjs5420939@gmail.com (J.S.P.); jhs0322@korea.kr (H.S.J.); 2 Research and Development Planning Division, National Institute of Fisheries Science, Busan 46083, Republic of Korea; jhlee@korea.kr

**Keywords:** eel (*Anguilla bicolor*), catfish (*Silurus asotus*), biofloc technology, aquaponics, FLOCponics

## Abstract

In this study, we sought to improve the productivity of Far Eastern catfish (*Silurus asotus*) and tropical eel (*Anguilla bicolor*), which are high-value fish species in the Republic of Korea, as well as that of associated crops by applying biofloc technology (BFT)-based aquaponics systems. The following three systems were used: the flow-through system (FTS), BFT, and BFT aquaponics system (BAPs). Caipira lettuce (*Lactuca sativa*) was utilized and hydroponics (HP) was implemented to compare crop productivity. After 42 days of treatment, the BAPs and BFT systems improved fish productivity, with weight gain rates of 134.47 ± 1.80% in BAPs-cat, 130.38 ± 0.95% in BFT, and 114.21 ± 6.62% in FTS for *S. asotus*, and 70.61 ± 3.26% in BAPs-eel, 62.37 ± 7.04% in BFT, and 47.83 ± 1.09% in FTS for *A. bicolor*. During the experiment, the total ammonia nitrogen and NO_2_^−^-N concentrations were stable in all plots. In the case of NO_3_^−^-N, BFT showed an increasing tendency while both BAPs showed a decrease compared with that of the BFT. BAPs-cat (total weight: 224.1 ± 6.37 g) and HP (220.3 ± 7.17 g) resulted in similar growth. However, in BAPs-eel was 187.7 ± 3.46 g due to root degradation. Water content analysis showed that BAPs-cat and BAPs-eel contained sufficient K, Ca, P, and S, which are important for crop growth. Overall, the effect of BAPs on fish growth was higher than that of FTS. This study reveals that integrating BFT with aquaponics improves productivity for high-value fish and associated crops while maintaining stable water quality. This method offers sustainable, efficient production, reduces environmental impact, and provides insights for future research in sustainable aquaculture practices.

## Simple Summary

This study aimed to evaluate the effects of a biofloc-based aquaponics system (FLOCponics) on the growth performance of two economically important fish species in Korea—Far Eastern catfish (*Silurus asotus*) and tropical eel (*Anguilla bicolor*)—applied with crop productivity using caipira lettuce (*Lactuca sativa*). Three systems were compared: a conventional flow-through system (FTS), a biofloc system (BFT), and a biofloc-based aquaponics system (BAPs). The BAPs improved fish growth and feed efficiency compared to the FTS, while also maintaining stable water quality. Additionally, crops grown in BAPs-cat showed comparable growth to those in hydroponics, suggesting strong synergy between fish and plant production. Although some root degradation occurred in BAPs-eel due to water characteristics, the system still supported healthy fish growth. These findings highlight FLOCponics as a promising sustainable method for indoor aquaculture and crop production, offering environmental and economic benefits.

## 1. Introduction

The aquaculture industry is influenced by the natural environment and uses large amounts of water [1]. Aquaculture farms are affected by environmental problems such as sewage, poor water quality, infections, and antibiotics overuse. Therefore, the demand for sustainable aquaculture systems is high. In particular, biofloc technology (BFT) and recirculating aquaculture systems (RASs) can reduce sewage by reusing rearing water [2,3,4]. Furthermore, BFT and RAS can decrease energy wastage and environmental problems caused by closed and indoor farming by removing nitrogen components (ammonia and nitrite) using a biological filtering system [5].

In a BFT, microorganisms (bioflocs) can reduce ammonia via heterotrophic bacteria dominance by controlling the carbon/nitrogen (C/N) ratio for ammonification [6]. Bioflocs can be used as additional feed for culturing species [7], thereby improving their immune system and growth [8,9,10]. However, low water exchange cannot reduce nitrate (NO_3_^−^), and floc overproduction has undesirable consequences, such as respiratory depression and rapid pH degradation, for species culturation [7,11,12] Recently, these problems have been addressed using aquaponics [13,14].

Aquaponic systems combine aquaculture and hydroponic systems [1]. Integrating the production of fish and plants is a currently expected practice. Aquaponics is an eco-friendly technology that primarily uses RAS combined with hydroponic systems and nutrients generated during fish rearing to grow crops [15,16,17,18]. Aquaponic systems have a substantial potential for producing proteins and crops for future industries. However, aquaponic systems have some limitations, including deficiencies in Ca, K, and Fe, which should be supplemented artificially [15]. Typically, 1 kg of fish can produce 20 individuals. Aquaponics primarily emphasizes plant rather than fish production due to the rapid harvesting of plants [19,20].

Generally, aquaponic systems operate as RAS. RAS with a coupled system has been shown to result in low nutrient concentrations [21] and crop productivity. To increase nutrient concentrations, decoupled aquaponic systems is recommended. However, decoupled aquaponic systems require more equipment and space for sump installation.

Recently, aquaponics based on BFT, also known as FLOCponics, has been developed and recommended to overcome the above issues [14,22]. BFT can effectively and quickly remove nitrogen compounds from feed debris and excrement using heterotrophic microorganisms with a high carbon/nitrogen (C/N) ratio to add carbon sources with low water exchange [4,6,7]. BFT also saves warming energy and blocks the inflow of diseases and parasites due to minimal rearing water exchange [11]. Carp, catfish, tilapia, and eel have been reportedly cultured in BFT [23]. BFT rearing water provides a source of nutrients for growing plants [24]. Electrical conductivity (EC) and nitrate (NO_3_^−^) are important indicators of plant growth due to the abundant organic matter in plants [25,26,27]. BFT is expected to serve as an additional biofilter in aquaponics because it provides high levels of EC and NO_3_^−^ for crop production [28,29,30].

This study aimed to determine the effect of applying BFT-based aquaponics to two types of fish, the Far Eastern catfish (*Silurus asotus*) and tropical eel (*Anguilla bicolor*), along with hydroponically grown caipira lettuce (*Lactuca sativa*). It also analyzes the elemental composition of rearing water to demonstrate the superiority of BFT water.

## 2. Materials and Methods

### 2.1. Systems

**Figure 1:**
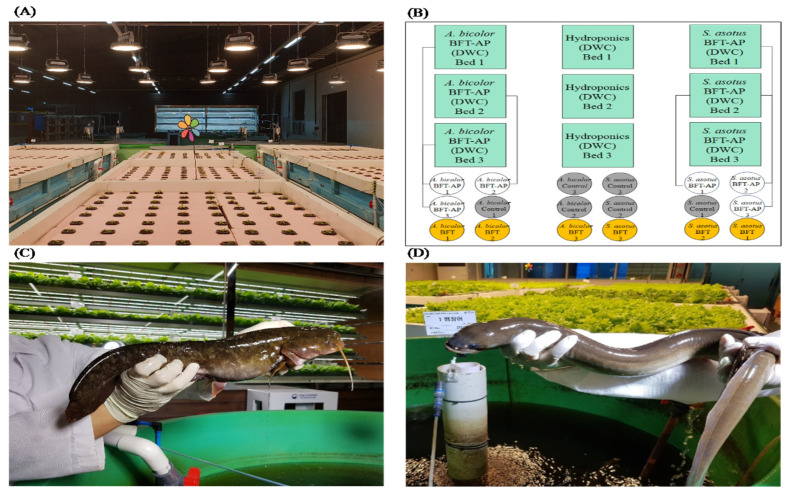
Preview and system view of experiments for Far Eastern catfish (*Silurus asotus*) and tropical eel (*Anguilla bicolor*). (**A**) Overview of experimental fish tank with crop beds; (**B**) ichnography of the BFT-based aquaponics system, Green: Plants bed, White: BAPs-, Yellow: BFT, Grey: Control; (**C**) Far Eastern catfish (*S. asotus*); (**D**) tropical eel (*A. bicolor*).

The experiment ran for 86 days (inoculation = 30 days, seedlings = 14 days, = fish and crop rearing = 42 days). Indoor catfish (cat)- and eel-rearing systems were implemented and classified into three types (Figure 1C,D): BFT, BFT with aquaponic systems (BAPs), and flow-through systems [FTSs (control): two rotations of water change/day (at 11:30 and 17:30)]. Two major types of aquaponic systems are available: deep water culture (DWC) and the nutrient film technique [31]. In this study, crop cultivation systems were combined with a DWC-coupled aquaponics system, which also included a hydroponics (HP) system as a control plot for crops produced using BAPs. The experiments consisted of 18 fiber-reinforced plastic (FRP) tanks (diameter 1.2 m × height 1.0 m) (in triplicate each for BFT-cat, BFT-eel, BAPs-cat, BAPs-eel, FTS-cat, and FTS-eel) for rearing fish and 9 crop FRP beds (width 2.0 m × length 2.0 m × height 0.8 m) (in triplicate each for BAPs and HP) for crop cultivation (Figure 1A,B).

### 2.2. Rearing and Water Management

**Figure 2:**
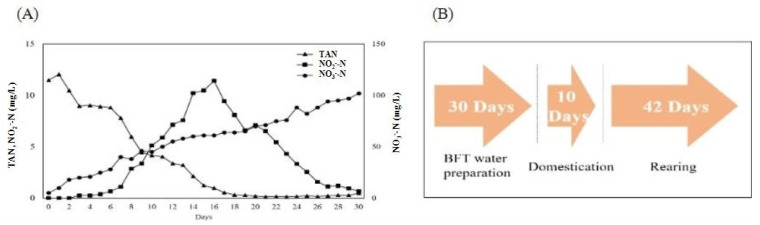
Nitrogen component variations of the BFT inoculating period (**A**) and total experimental process and days (Arrow indicate the order of the experiment. First, 30 days; BFT water preparation, Second, 10 days; Domestication, Final, 42 days; Rearing) (**B**).

BFT rearing was performed as described by former research [32]. Briefly, 20 t of water was inoculated by adding BFT-ST (Bacteria seed, EcoTech Service, Gimpo-si, Republic of Korea) at 100 ppm, along with commercial feed (crude protein: 44%, Sajo Dong-A One, Seoul, Republic of Korea) as a nitrogen source, at a rate of 5 kg/day for approximately 1 month. By maintaining a C/N ratio of 15:1, refs. [6,11] calculated the carbon demand by adding molasses (Carbon: 50.08%). Water temperature was maintained at approximately 25.0 °C using a 1 kW heater (OKE-HE-100, SEWON OKE, Suwon, Republic of Korea). At the end of inoculation, the TAN was 0.482 mg/L, NO_2_^−^-N was 0.683, NO_3_^−^-N was 88 mg/L, and the Imhoff cone measurement was 15.5 mL/L (Figure 2). After inoculation (when the TAN and NO_2_^−^-N reached the maximum values and decreased to approximately 1 mg/L), the fish were acclimated by feeding for 10 days to start the experiment with 20% of rearing freshwater.

After acclimation, the fish were grouped, and water was distributed to each fish tank and plant bed. During the rearing period, the C/N ratio of the BFT and BAPs was maintained at 15:1. The BFT and BAPs were strongly aerated to prevent the settlement of bioflocs using an air blower (Hi-blow HP-80, Techno Takatsuki Co., Ltd., Osaka, Japan) and a water pump (25 W; Hyupsin, Seoul, Republic of Korea). An oxygen supply machine (KMOS-40Rl, Kumho Marine, Busan, Republic of Korea) was used to supply sufficient oxygen for FTS. Each crop bed was weakly aerated to prevent floc deposition on crop roots. Additionally, the BAPs were installed with drainage pipes to provide water to the crop beds, and the water quantity was set to two cycles (input volume from the fish tank) per day. No water changes were made in the BFT and BAPs.

### 2.3. FLOCponics and Hydroponics

The crop cultivation systems were divided into three types (BAPs-catfish, BAPs-eel, and HP). Each type included triplicate setups. All crop beds were subjected to DWC and comprised 90 pots per bed to cultivate the crops and a coupled system, as described by [21]. Artificial lighting was installed for indoor crop cultivation, and the 12 L:12 D conditions adopted >6000 lx (daily average) for photosynthesis. HP was conducted as a control for crop cultivation and commercial artificial nutrient solutions (Liquid A: N 2%, K 3.5%, Ca 2%, Fe 0.05%; Liquid B: 1.3%, P 1.5%, K, 5%, Mg 0.7%, B 0.05%, Mn 0.01%, Zn 0.002%) (Mulfuresiriz, Daeyu Business Limited, Seoul, Republic of Korea) were used at 1000 ppm by manual.

Caipira lettuce was selected for crop cultivation. Lettuce is typically grown in cool environments. Caipira lettuce seeds (Enza Zaden, Enkhuizen, The Netherlands) grafted onto terra-plugs (Smithers-Oasis Co., Ltd., Kent, OH, USA) were germinated in a germination chamber (indoor temperature; 24 °C) at the Advanced Aquaculture Research Center. After 14 days of germination, the seedlings reached the true-leaf stage (4 days post-germination), and 90 seedlings (2.0 ± 0.47 g) were transplanted per bed.

### 2.4. Fish

A total density of 5.75 ± 0.005 kg (5 kg/m^2^) for Far Eastern catfish (mean body weight: 61.6 ± 5.91 g) and tropical eel (mean body weight: 83.8 ± 6.89 g) was used for the experiment. The fish were acclimated and cultured at the Advanced Aquaculture Research Center, Changwon, Republic of Korea, until maximum crop growth was reached (approximately 30 days after transplanting) [33].

The commercial feed (extruded pellets) used was obtained from Sajo Dong-A One and contained 44% crude protein (CP), crude lipid, 1% calcium, 14% moisture, 1.8% phosphate, 5% crude fiber, and 17% ash. Generally, eels consume high-protein feed (over 50% CP), but both fish species were given the same feed to ensure the analysis of the breeding water contents was not influenced. Feed was provided to full satiety for Far Eastern catfish (approximately 5% of total weight/day) and tropical eels (approximately 3% of total weight/day) thrice daily (09:30, 13:00, and 16:30) at the concentration of each biomass for 6 weeks. Molasses (carbon source) was added to the final feeding after 1 h at 17:30. Water temperature was maintained at approximately 25.0 °C using a 1-kW heater (OKE-HE-100, Sewon OKE, Suwon, Republic of Korea).

### 2.5. Growth Performance and Production of Fish and Crops

At the conclusion of the 4th week of the experiment, the growth performance and production metrics of each experimental group of fish and crops were evaluated.

The water conditions in the tanks were assessed using a YSI-650 Multiparameter Display System (YSI Incorporated, Yellow Springs, OH, USA) for the following parameters: temperature (°C), dissolved oxygen (DO, mg/L), pH, EC (dS/cm), and total dissolved solids (TDS, mg/L). The breeding water in all plots was sampled daily at AM 09:30 before feeding. The pH was controlled using sodium bicarbonate when the pH was lower than 6.5. The nitrogen components, including total ammonia nitrogen (TAN), nitrite nitrogen (NO_2_^−^-N), and nitrate nitrogen (NO_3_^−^-N), were measured using an analysis kit (Merck KGaA, Darmstadt, Germany) and an absorptiometer (Merck KGaA), employing the three dust spot methods. At the end of the experiment, rearing water from Far Eastern catfish, tropical eel, and the HP system was preserved at −80 °C. Subsequently, 500 mL of rearing water was analyzed (mixed with each plot bed). The analysis included the total nitrogen measurement using a Kjeltec 2300 nitrogen analyzer (FOSS Tecator, Hillerød, Denmark), the total phosphate using a UV2450 spectrophotometer (Shimadzu, Kyoto, Japan), and K, Ca, Mg, Fe, Cu, Zn, and Si concentrations using an Optima 8300 inductively coupled plasma optical emission spectrometer (PerkinElmer. Waltham, MA, USA). Cl and SO_4_ were analyzed using ion chromatography (930 Compact IC Flex; Metrohm Co., Herisan, Switzerland).

The growth performance and production of each experimental fish and crop group were measured at the end of the study period. Thirty fishes were randomly sampled from each tank and anesthetized using 100 ppm MS-222 (Sigma Aldrich, St. Louis, MO, USA) to measure their body weight and total length. Growth performance factors were calculated using the following equations:

Weight gain rate (WGR, %) = (Final weight − Initial weight)/(Initial weight) × 100

Specific growth rate (SGR, %/day) = [ln (final body weight) − ln (initial body weight)]/day × 100

Feed conversion ratio (FCR) = (supplied feed)/(final weight − initial weight)

Survival rate (%) = (Final individuals/initial individuals) × 100

After 6 weeks, 10 crops from each bed were randomly sampled from each experimental group, and the total weight and length, shoot weight and length, leaf length and width, and number of leaves were recorded.

An electronic scale (MW-200; CAS, Seoul, Republic of Korea) was used to measure body weight to an accuracy of 0.01 g, and Vernier calipers (Mitutoyo Electronic, Kawasaki, Japan) were used to measure the total length with a precision of 0.1 mm.

### 2.6. Stress Parameters

Serum stress parameters indicate the health of freshwater fish [34]. Fish were randomly sampled from each experimental group, anesthetized using 100 ppm MS-222 (Sigma Aldrich), and blood was drawn from the caudal vein using 20 IU/mL heparin (Sterile Solution HEPARIN Inj.; Choongwae Pharma Corporation, Seoul, Republic of Korea)-treated syringes (3 mL; Dong Shin Medical Instruments Co., Miryang, Republic of Korea). Thereafter, the samples were centrifuged at 2339× *g* and 4 °C for 15 min (Smart R17 Plus; Hanil Co., Dangjin, Republic of Korea) to obtain plasma, which was stored at −80 °C until use. Subsequently, plasma glucose (GLU), glutamate–pyruvate transaminase (GPT), and glutamate–oxaloacetate transaminase (GOT) levels were analyzed using an automatic dry chemistry analyzer (Fuji Dri-Chem NX600V, Fujifilm Corporation, Tokyo, Japan), and cortisol levels were determined using an ELISA kit (Mybiosource, San Diego, CA, USA).

### 2.7. Statistical Analysis

Fish growth performance and crop production were analyzed using one-way analysis of variance in SPSS (version 22.0; SPSS Inc., Chicago, IL, USA), and differences between treatment groups were assessed via Tukey’s test. *p* < 0.05 was considered statistically significant.

## 3. Results

### 3.1. Water Quality

The water conditions in each fish tank were consistently maintained throughout the experiment. The water parameters for the BAPs-cat, BFT-cat, and FTS experimental groups were as follows: temperature (°C) = 25.19 ± 0.53, 25.70 ± 0.42, and 25.76 ± 0.42; DO (mg/L) = 11.44 ± 1.51, 11.16 ± 1.39, and 11.12 ± 0.64 mg/L; pH = 7.15 ± 0.20, 7.25 ± 0.30, and 7.09 ± 0.27; EC (dS/cm) = 0.78 ± 0.05, 1.30 ± 0.21, and 0.20 ± 0.04; TDS (mg/L) = 0.39 ± 0.02, 0.66 ± 0.11, and 0.10 ± 0.01, respectively. The BAPs-eel, BFT-eel, and FTS experimental groups were as follows: temperature (°C) = 25.55 ± 0.45, 25.67 ± 0.43, and 25.21 ± 1.91; DO (mg/L) = 11.29 ± 1.86, 11.36 ± 1.09, and 11.13 ± 0.85 mg/L; pH = 7.04 ± 0.25, 6.48 ± 0.84, and 7.19 ± 0.11; EC (dS/cm) = 1.53 ± 0.02, 1.86 ± 0.26, and 0.25 ± 0.02; TDS (mg/L) = 0.77 ± 0.01, 0.94 ± 0.13, and 0.12 ± 0.02, respectively (Table 1).

**Figure 3:**
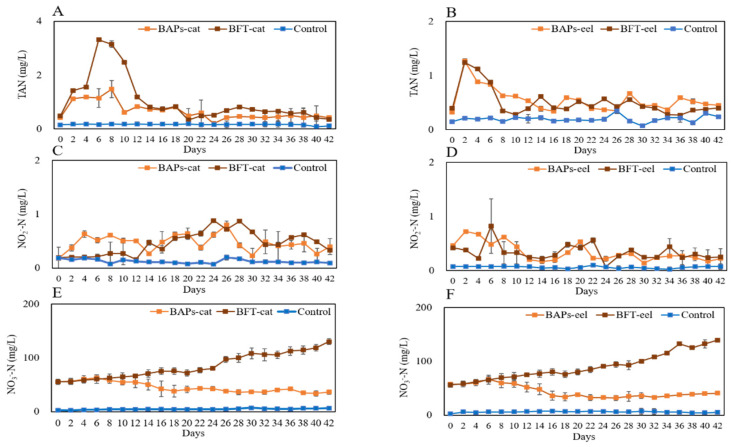
Water quality variations of BAPs, BFT, and FTS systems containing catfish and eel for 6 weeks ((**A**,**C**,**E**) for catfish, (**B**,**D**,**F**) for eel).

The nitrogen parameters for the BAPs-cat, BFT-cat, and FTS experimental groups were as follows: TAN = 0.75 ± 0.353, 1.26 ± 0.970, and 0.17 ± 0.011 mg/L and NO_2_^−^-N = 0.50 ± 0.160, 0.44 ± 0.254, 0.13 ± 0.042 mg/L, respectively. The parameters were stabilized at an average of approximately 1 mg/L during the experiment (Figure 3A,C). The NO_3_^−^-N levels were highest in BFT (day 28, 100.10 ± 8.480 mg/L), followed by BAPs (day 6, 63.12 ± 5.500 mg/L) and the control (day 16, 7.11 ± 1.220 mg/L). In BAPs, the NO_3_^−^-N levels decreased after day 6 (Figure 3E). The BAPs-eel, BFT-eel, and FTS experimental groups were as follows: TAN = 0.58 ± 0.259, 0.57 ± 0.286, and 0.20 ± 0.049 mg/L and NO_2_^−^-N = 0.39 ± 0.184, 0.36 ± 0.175, 0.06 ± 0.017 mg/L, respectively. The parameters were stabilized at an average below 1 mg/L during the experiment (Figure 3B,D). NO_3_^−^-N levels were highest in BFT (day 28, 139.48 ± 8.480 mg/L), followed by BAPs (day 6, 66.54 ± 5.540 mg/L) and the control (day 16, 7.66 ± 1.220 mg/L) (Figure 3F). In BAPs, NO_3_^−^-N levels decreased after day 6 but increased in BFT, similar to catfish.

### 3.2. Fish Growth

Both catfish and eels showed higher growth in BAPs and BFT than in FTS. The growth performance of catfish is presented in Table 2. The tank final weight, WGR, FCR, and SGR were higher in the BAPs group (13.48 ± 0.10 kg, 83.83% ± 1.55%, 1.04 ± 0.01, and 2.95 ± 0.12%/day, respectively), followed by the BFT (13.25 ± 0.06 kg, 78.66 ± 0.84%, 1.07 ± 0.01, and 2.94 ± 0.02%/day, respectively) and control (12.32 ± 0.38 kg, 72.72 ± 5.03%, 0.23 ± 0.07, 2.78 ± 0.14%/day) groups (Tukey’s test, *p* < 0.05).

The growth performance of the eels showed a similar tendency. The tank final weight, WGR, FCR, and SGR were higher in the BAPs group (9.81 ± 0.19 kg, 70.61% ± 3.26%, 1.33 ± 0.06, and 1.82 ± 0.03%/day, respectively), followed by the BFT (9.34 ± 0.41 kg, 62.38 ± 7.04%, 1.52 ± 0.18, and 1.62 ± 0.06%/day, respectively) and control (8.50 ± 0.06 kg, 47.83 ± 1.09%, 1.96 ± 0.04, 1.46 ± 0.10%/day) groups (Tukey’s test, *p* < 0.05). The 100% survival rate is shown in each plot (Table 2).

### 3.3. Crop Growth

**Figure 4:**
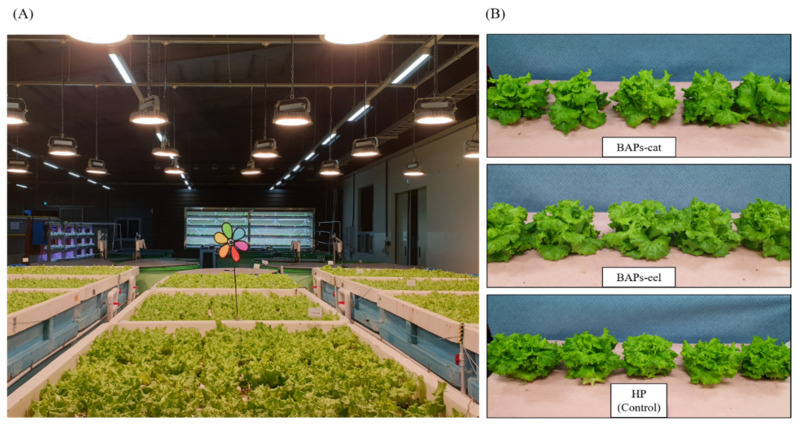
Overview of caipira lettuce in BAPs-cat, BAPs-eel, and HP (Control) beds 6 weeks after planting seedlings (**A**) and crop view of BAPs-cat, BAPs-eel, and HP (Control) (**B**).

Figure 4 presents an overview at the conclusion of the rearing experiment. The total length (557.03 ± 8.33 mm) and weight (187.73 ± 3.63 g) of caipira lettuce in BAPs-eel were lower than those in the other two systems due to root degradation (BAPs-cat, 594.07 ± 19.53 mm, 224.11 ± 6.37 g; HP, 596.15 ± 52.62 mm, 220.28 ± 7.17 g, respectively).

However, the other parameters were similar for all three systems, as follows: shoot length: BAPs-cat, 219.97 ± 6.20 mm; BAPs-eel, 219.48 ± 10.20 mm; HP, 215.48 ± 11.33 mm; shoot weight: BAPs-cat, 186.28 ± 6.02 g; BAPs-eel, 182.12 ± 3.33 g; HP, 186.13 ± 7.24 g; leaf width: BAPs-cat, 211.88 ± 9.89 mm, BAPs-eel, 212.08 ± 6.44 mm, HP, 209.22 ± 12.53 mm; leaf length: BAPs-cat, 157.45 ± 12.42 mm, BAPs-eel, 156.09 ± 15.27 mm, HP, 155.01 ± 21.83 mm; number of leaves: BAPs-cat, 26 ± 1; BAPs-eel, 27 ± 2; HP, 26 ± 1 (*p* ≥ 0.05) (Table 3). However, except for the total weight and length in BAPs-eel, these parameters showed that the caipira lettuce production in the two BAPs systems was similar to that in the HP system.

### 3.4. Stress Analysis

The hematologic responses of catfish and eel to each method are presented in Table 4. Hematologic and serum parameters indicate the health and nutritional status of organisms [35]. The AST/GOT, ALT/GPT, and GLU levels did not significantly differ between the systems. Additionally, catfish showed higher AST/GOT and ALT/GPT ratios than eels but higher GLU than eels.

### 3.5. Rearing Water Content Composition

K, Ca, P, and S are the most important mineral components for plant growth [36]. K was highest in HP (61.01 mg/L), followed by BFT-eel (30.75 mg/L), BFT-catfish (20.48 mg/L), BAPs-eel (14.11 mg/L), BAPs-cat (13.43 mg/L), FTS-cat (2.43 mg/L), and FTS-eel (2.32 mg/L). Ca was highest in BFT-eel (121.85 mg/L), followed by BFT-cat (117.33 mg/L), HP (114.85 mg/L), BAPs-eel (51.04 mg/L), BAPs-cat (47.33 mg/L), FTS-eel (31.26 mg/L), and FTS-cat (24.54 mg/L). P was highest in HP (28.13 mg/L), followed by BFT-eel (25.93 mg/L), BAPs-eel (18.13 mg/L), BFT-cat (15.21 mg/L), BAPs-cat (8.07 mg/L), FTS-eel (4.40 mg/L), and FTS-cat (3.47 mg/L). S was highest in BFT-eel (131.19 mg/L), followed by HP (124.11 mg/L), BFT-cat (111.45 mg/L), BAPs-eel (75.16 mg/L), BAPs-cat (51.45 mg/L), FTS-cat (12.05 mg/L), and FTS-eel (11.64 mg/L) (Table 5).

Notably, Na was higher in BFT-eel (108.05 mg/L) and BAPs-eel (75.95 mg/L) than in the other systems. Other minerals were highest in HP, followed by BFT, BAPs, and FTS.

## 4. Discussion

Water can be used to create suitable growth environments for fish and vegetable cultivation [37]. While a C/N ratio of 15:1 or more in BFT water can facilitate the dominance of heterotrophic bacteria (*Bacillus* sp., *Cellulomonas* sp.), a C/N ratio under 15:1 could facilitate the dominance of autotrophic bacteria (*Nitrosomonas* sp. *Nitrobacter* sp.) [4,38].

Over the 30-day inoculation period, fluctuations were observed with increases and decreases in TAN and NO_2_^−^-N, along with an increase in NO_3_^−^-N. These concentration levels indicated that feeds could sufficiently sustain bacterial growth and activity.

Ammonia decomposition occurs through three processes: autotrophic decomposition by phytoplankton, nitrification by autotrophic bacteria, and ammonification by heterotrophic bacteria [39,40,41]. Over the 42-day rearing period, the TAN levels in both BFT and BAPs increased during the first week but gradually diminished and stabilized under 1 mg/L by the end of the experiment.

Heterotrophic bacteria dominance in the BAPs and BFT increased, and flocs were formed in the early phase, indicating that in the first week, bacteria were less abundant than the input from fish. The feces of rearing fish were released into BFT water, enabling heterotrophic bacteria proliferation with a decrease in TAN [42]. The input of feed and molasses to maintain the C/N ratio could provide adequate conditions for fish rearing. Rearing water with a stable quality results in low fish mortality [43]. When the pH is low, high NO_2_^−^-N concentrations damage the fish, resulting in high mortality. However, in the present study, high NO_2_^−^-N concentrations were not observed (<1 mg/L). In the case of NO_3_^−^-N, FTS showed low concentrations while BFT showed increased concentrations until the end of the experiment. In contrast, in both BAPs-cat and BAPs-eel, the NO_3_^−^-N concentrations decreased after 1 week of rearing. NO_3_^−^ is a source of nutrients for crops [44]. Therefore, the decreasing tendency of NO_3_^−^-N in BAPs than in BFT indicates that BFT has high nutrient concentrations. This suggests that crops can be used as biological filter tools for nitrogen [45].

The growth performance of catfish and eel in both BAPs and BFT was superior to that in FTS. According to previous studies, BFT positively affects feed efficiency in African catfish (*Clarias gariepinus*) [46], European eel (*A. anguilla*) [47], and other species [48]. Biofloc provides an additional source of nutrients for fish [49] by decomposing ammonia into amino acids using carbon sources (ammonification) and partially decomposing nitrogen components through nitrification [6]. The current study showed that 1 kg of feed was conserved by BAPs and BFT compared with that in FTS, and the catfish culture in BAPs and BFT saved more energy than did the eel culture [38]. All tanks showed > 90% survival rates, with most catfish being of similar size. However, some abnormally growing fish appeared, leading to incidents of cannibalism. No cannibalism was observed in the eel plots. Reducing cannibalism in catfish culture in BAPs and BFT could result in better productivity relative to FTS.

At the end of the experiment, similar growth was observed in BAPs-cat, BAPs-eel, and caipira lettuce HP. The number of edible portions (shoots, i.e., leaves and stems) was similar in each plot. However, BAPs-eel had the lowest total plant weight and length among the three systems. Compared with those in the other two plots, no damaged individuals were observed in the BAPs-catfish and HP plots, but the roots were damaged and shortened in the BAPs-eel plot. Roots allow plants to absorb minerals and nutrients, with their absorbance capacity depending on the root surface area [50]. In aquaponics based on BFT, root degradation is caused by the amounts of flocs and solids [14].

In this study, decoupled FLOCponics with solid removal and flow rate control is recommended in BAPs-eel, and reducing the flow rate can reduce the high floc and solid concentrations in the plant bed. However, lower flow rates cause clogging of the pipes by flocs and solids. Our results showed that only one system cannot be applied to all fish species, and it would be better to supplement the characterized system to increase economic feasibility depending on the fish species.

The causes of stress are distinguished based on the physical, chemical, and biological effects of external and internal factors [51]. GOT, GPT, and GLU are functional health and metabolic biomarkers in aquatic animals [52]. In the present study, the concentrations of AST/GOT, ALT/GPT, GLU, and cortisol in all fish and systems did not significantly differ (*p* > 0.05). Cortisol concentration is related to many stress processes, metabolism, osmoregulation, and feeding behavior [53,54].

Regardless of how well designed current systems are, they should cause less stress and provide better welfare for cultured species than the old methods. A previous study showed that ALT/GPT (U/L) was 6.3 ± 0.5, AST/GPT (U/L) was 153.7 ± 16.2, and GLU (mg/dL) was 67.7 ± 29.0 in *S. asotus* [55]. Japanese eel (*A. japonica*) showed ALT/GPT (U/L), AST/GOT (U/L), and GLU (mg/dL) of <10 (U/L), 105.6 ± 38.3 (U/L) and 164.8 ± 45.7 (mg/dL), respectively, healthy *A. japonica* in freshwater culture [56].

The stress parameters and survival rates showed that BAPs and BFT provided adequate and suitable conditions for rearing *S. asotus* and *A. bicolor*. However, as the rearing period increased, the size of the fish gradually increased, and the food supply increased accordingly. This can lead to higher nutrient concentrations in BFT water over time, resulting in an inappropriate environment for fish and crops. Therefore, as the rearing period increases, modifying and maintaining a high producible density and continuously monitoring water quality and mineral component variations are important.

Generally, RAS-based aquaponics produces adequate amounts of NO_3_^−^ to meet plant requirements. Nevertheless, most aquaponic systems undergo a lack of K, Ca, P, and S. Additionally, RAS-based aquaponics requires high concentrations of artificial nutrient solutions, resulting in eutrophication due to sewage from aquaponic beds [57]. In a BFT system, the addition of organic carbon sources is vital for maintaining the C/N ratio. BFT water can be produced using feed and molasses; other carbon sources, such as glucose and glycerol, are more expensive than molasses. Minerals in molasses comprise K (1.960%), Na (0.120%), Ca (0.52%), Mg (0.20%), P (0.78%), and Fe (0.02%). This enriched nutrient concentration in BFT water could replace artificial nutrient solutions [14,58].

According to Yang and Kim, 2020 [33], the important element K, P, and S were found to be higher compared to this study, with concentrations of 75.2 mg/L, 30.1 mg/L, and 242.3 mg/L, respectively, whereas Ca was lower at 18.4 mg/L. The deficiency of K, Ca, P, and S in plantation systems results in poor root and leaf growth, chlorosis, and increased susceptibility to diseases [59]. In the present study, the water content in BAPs-eel was adequate and higher than that in BAPs-cat. Nevertheless, the lettuce in the BAPs-eel system showed damaged and shortened roots compared with those in the other systems.

During the rearing period, the pH in BAPs-eel and BFT-eel decreased faster than that in BAPs-cat and BFT-cat. The low pH in eel systems requires more sodium bicarbonate to increase the pH. This leads to high Na concentrations in BAPs-eel and BFT-eel. The lettuce in BAPs-eel was influenced by this Na concentration, resulting in shortened roots [60,61].

Therefore, in eel aquaponics, conducting further research on optimal stocking density and water quality management is necessary to reduce the use of sodium bicarbonate to control Na concentrations. Despite the lower content of BAPs-cat compared to BAPs-eel, *S. asotus* is more adaptable than *A. bicolor* for crop growth in BAPs with this experimental design. These results indicate that combining fish species and crops can influence BAPs productivity. The most important aspects of aquaponics are its high productivity, lack of fish and crop deterioration, and system sustainability.

For example, fish cultured in BFT with high mineral concentrations would be well suited for nutrient-demanding crops such as basil (*Ocinum basilicum*) [13] and cherry tomato (*Solanum lycopersicum*) [62]. BAPs-eel resulted in a higher concentration and degradation of roots than BAPs-cat. To enhance the productivity and economic feasibility of *A. bicolor*, reforms in BAPs-eel should include increasing the number of crops and crop beds, lowering eel density, or adopting decoupled FLOCponics to improve the mineral absorbing capacity and decrease root degradation.

Water pollution and scarcity and agricultural land degradation have increased [63]. Aquaponic systems can be applied to several crops and fish species to solve water availability problems caused by radical changes in the global environment and climate. Although our results show that BAPs could give adequate for fish and crop growth, further studies are necessary to refine the operations. Achieving sustainable culturing will be possible by determining the optimal combination of composition, density, and environment through studies of crop and fish species, as well as gut and water microbiota.

## 5. Conclusions

FLOCponics, which integrates biofloc technology with aquaponics, significantly enhanced the growth of fish and the productivity of lettuce. Compared to traditional systems, it provided superior water quality, nutrient availability, and feed conversion efficiency. While the BAPs-cat system was successful, root degradation in the BAPs-eel system indicated that species-specific adjustments are necessary. The study concludes that FLOCponics is a viable and eco-friendly alternative for sustainable food production, recommending future research to focus on system refinement and species compatibility.

## Figures and Tables

**Table 1:** Water condition of *S. asotus* and *A. bicolor* in BAPs, BFT, and FTS for 6 weeks.

Systems	Parameters				
	Temp (°C)	DO (mg/L)	pH	EC (dS/cm)	TDS (mg/L)
*S. asotus*					
BAPs-cat	25.19 ± 0.53 ^ns^	11.44 ± 1.51 ^ns^	7.15 ± 0.20 ^ns^	0.78 ± 0.05 ^b^	0.39 ± 0.02 ^b^
BFT-cat	25.70 ± 0.42	11.16 ± 1.39	7.25 ± 0.30	1.30 ± 0.21 ^a^	0.66 ± 0.11 ^a^
FTS-cat	25.76 ± 0.42	11.12 ± 0.64	7.09 ± 0.27	0.20 ± 0.04 ^c^	0.10 ± 0.01 ^c^
*p*-value	*p* ≥ 0.05	*p* ≥ 0.05	*p* ≥ 0.05	*p* < 001	*p* < 0.001
*A. bicolor*					
BAPs-eel	25.55 ± 0.45^ns^	11.29 ± 1.86 ^ns^	7.04 ± 0.25 ^ns^	1.53 ± 0.02 ^a^	0.77 ± 0.01 ^a^
BFT-eel	25.67 ± 0.43	11.36 ± 1.09	6.48 ± 0.84	1.86 ± 0.26 ^a^	0.94 ± 0.13 ^a^
FTS-eel	25.21 ± 1.91	11.13 ± 0.85	7.19 ± 0.11	0.25 ± 0.02 ^b^	0.12 ± 0.02 ^b^
*p*-value	*p* ≥ 0.05	*p* ≥ 0.05	*p* ≥ 0.05	*p* < 0.001	*p* < 0.001

Data presented as a mean ± S.D. The data in rows denoted with different letters are statically different (*p* < 0.05). ns, no significant (*p* ≥ 0.05).

**Table 2:** Growth performance of *S. asotus* and *A. bicolor* in BAPs, BFT, and FTS for 6 weeks.

Parameters	*S. asotus*				*A. bicolor*			
	BAPs-Cat	BFT-Cat	FTS-Cat	*p*-Value	BAPs-Eel	BFT-Eel	FTS-Eel	*p*-Value
^1^ FBW (g)	145.39 ± 20.34 ^a^	140.22 ± 15.82 ^a^	134.28 ± 15.49 ^b^	*p* < 0.001	139.36 ± 10.97 ^a^	132.03 ± 6.42 ^ab^	125.93 ± 13.48 ^b^	*p* < 0.001
^2^ FTW (kg)	13.48 ± 0.10 ^a^	13.25 ± 0.05 ^a^	12.32 ± 0.38 ^b^	*p* = 0.0018	9.81 ± 0.18 ^a^	9.34 ± 0.41 ^ab^	8.50 ± 0.06 ^b^	*p* = 0.0084
^3^ WGR (%)	134.47 ± 1.80 ^a^	130.38 ± 0.95 ^a^	114.21 ± 6.62 ^b^	*p* = 0.0118	70.61 ± 3.26 ^a^	62.37 ± 7.04 ^ab^	47.83 ± 1.09 ^b^	*p* = 0.0024
^4^ SGR (%/day)	2.05 ± 0.03 ^a^	1.96 ± 0.01 ^a^	1.86 ± 0.03 ^b^	*p* = 0.0136	1.21 ± 0.02 ^a^	1.08 ± 0.06 ^ab^	0.97± 0.07 ^b^	*p* = 0.0025
^5^ FCR	1.12 ± 0.02 ^a^	1.15 ± 0.01 ^a^	1.29 ± 0.03 ^ab^	*p* = 0.0039	1.33 ± 0.06 ^a^	1.51 ± 0.18 ^ab^	1.96 ± 0.04 ^b^	*p* = 0.0013
^6^ SR (%)	95.4 ± 1.90 ^ns^	95.4 ± 3.72	92.6 ± 2.05	*p* ≥ 0.05	100.0 ± 0.0 ^ns^	100.0 ± 0.0	100.0 ± 0.0	*p* ≥ 0.05

^1^ FBW: Final body weight, ^2^ FTW: Final total weight, ^3^ WGR: Weight gain rate, ^4^ SGR: Specific growth rate, ^5^ FCR: Feed conversion ratio, ^6^ SR: Survival rate. Data presented as a mean ± S.D. The data in rows denoted with different letters are statically different (*p* < 0.05). ns, no significant (*p* ≥ 0.05).

**Table 3:** Comparison of caipira lettuce (*L. sativa*) in BAPs-cat, BAPs-eel, and hydroponics (HP) for 6 weeks.

Parameters	Systems			
	BAPs-Cat	BAPs-Eel	Hydroponics (Cont.)	*p*-Value
Total length (mm)	594.07 ± 19.53 ^a^	557.03 ± 8.33 ^b^	596.15 ± 52.62 ^a^	*p* < 0.001
Shoot length (mm)	219.97 ± 6.20 ^ns^	219.48 ± 10.20	215.48 ± 11.33	*p* ≥ 0.05
Total weight (g)	224.11 ± 6.37 ^a^	187.73 ± 3.63 ^b^	220.28 ± 7.17 ^a^	*p* < 0.001
Shoot weight (g)	186.28 ± 6.02 ^ns^	182.12 ± 3.33	186.13 ± 7.24	*p* ≥ 0.05
Leaf width (mm)	211.88 ± 9.89 ^ns^	212.08 ± 6.44	209.22 ± 12.53	*p* ≥ 0.05
Leaf length (mm)	157.45 ± 12.42 ^ns^	156.09 ± 15.27	155.01 ± 21.83	*p* ≥ 0.05
No. leaves	26 ± 1 ^ns^	27 ± 2	26 ± 1	*p* ≥ 0.05

Data presented as a mean ± S.D. The data in rows denoted with different letters are statically different (*p* < 0.05). ns, no significant (*p* ≥ 0.05).

**Table 4:** Plasma chemistry parameters of *S. asotus* and *A. bicolor* in BAPs, BFT, and FTS for 6 weeks.

Systems	Parameters			
	AST/GOT (U/L)	ALT/GPT (U/L)	GLU (mg/dL)	Cortisol (ng/mL)
*S. asotus*				
BAPs-cat	62.27 ± 4.09 ^ns^	15.98 ± 1.19 ^ns^	62.03 ± 3.17 ^ns^	0.17 ± 0.11 ^ns^
BFT-cat	63.64 ± 5.52	16.17 ± 1.38	62.93 ± 4.39	0.13 ± 0.05
FTS-cat	62.97 ± 6.08	16.48 ± 1.48	62.31 ± 5.14	0.19 ± 0.11
*p*-value	*p* ≥ 0.05	*p* ≥ 0.05	*p* ≥ 0.05	*p* ≥ 0.05
*A. bicolor*				
BAPs-eel	50.37 ± 6.33 ^ns^	8.67 ± 1.18 ^ns^	152.13 ± 13.81 ^ns^	0.81 ± 0.30 ^ns^
BFT-eel	49.83 ± 8.23	8.97 ± 1.03	153.83 ± 15.10	0.79 ± 0.25
FTS-eel	49.67 ± 5.36	8.97 ± 1.40	151.43 ± 21.33	0.81 ± 0.30
*p*-value	*p* ≥ 0.05	*p* ≥ 0.05	*p* ≥ 0.05	*p* ≥ 0.05

Data presented as a mean ± S.D. ns, no significant (*p* ≥ 0.05).

**Table 5:** Water content analysis of BAPs-cat, BAPs-eel, and hydroponics for 6 weeks.

Contents	Systems						
	BAPs-Cat	BFT-Cat	FTS-Cat	BAPs-Eel	BFT-Eel	FTS-Eel	HP
Total-N (mg/L)	71.34	129.18	32.33	93.17	160.53	34.55	323.48
Total-P (mg/L)	8.07	15.21	3.47	18.13	25.93	4.40	28.13
Na (mg/L)	35.66	52.21	16.46	75.95	108.05	22.21	31.91
K (mg/L)	13.43	20.48	2.43	14.11	30.75	2.32	61.01
Ca (mg/L)	47.33	117.33	24.54	51.04	121.85	31.26	114.85
Mg (mg/L)	8.83	10.24	5.14	8.47	20.78	6.51	31.71
Fe (mg/L)	0	0	0	0	0	0	0.01
Mn (mg/L)	0	0	0	0	0	0	0
Zn (mg/L)	0.16	0.20	0	0.24	0.38	0.01	0.25
Cu (mg/L)	0.03	0.03	0.01	0.10	0.08	0.05	0.02
S (mg/L)	51.45	111.45	12.05	75.16	131.19	11.64	124.11
Cl (mg/L)	30.35	32.52	18.41	31.37	32.04	20.12	29.00
Si (mg/L)	13.42	13.69	13.26	15.09	32.04	13.28	22.99

## Data Availability

The original contributions presented in this study are included in the article. Further inquiries can be directed to the corresponding author.
